# Application of Building Information Modelling (BIM) in the Health Monitoring and Maintenance Process: A Systematic Review

**DOI:** 10.3390/s21030837

**Published:** 2021-01-27

**Authors:** Reihane Shafie Panah, Mahdi Kioumarsi

**Affiliations:** 1 International Institute of Earthquake Engineering and Seismology, Tehran 19537-14453, Iran; reihane.shafie@alumni.iiees.ac.ir; 2 Department of Civil Engineering and Energy Technology, OsloMet—Oslo Metropolitan University, 0167 Oslo, Norway

**Keywords:** building information modelling, structural health monitoring, BIM, SHM, sensor, maintenance, health assessment, structural health control, structural monitoring, bridge

## Abstract

Improvements in the science of health monitoring and maintenance have facilitated the observation of damage and defects in existing structures and infrastructures, such as bridges and railways. The need to extend sensing technology through the use of wireless sensors as well as the lack of description tools for understanding, visualizing, and documenting sensor outputs has encouraged researchers to use powerful tools such as Building Information Modelling (BIM) systems. BIM has become important because of conducting tools widely used in the Architecture, Engineering, and Construction (AEC) industry to present and manage information on structural systems and situations. Since combining health monitoring and maintenance results with BIM models is a new field of study, and most projects utilize various aspects of it, we have conducted a review of important work related to this subject published from 2010 to November of 2020. After reviewing 278 journal articles, research trends, approaches, methods, gaps, and future agenda related to BIM in monitoring and maintenance were highlighted. This paper, through a bibliometric and content analysis, concludes that besides main improvements, some limitations now exist which affect the modeling and maintenance process. These limitations are related to extending the IFC schema, optimizing sensor data, interoperability among various BIM platforms, optimization of various sensing technologies for fault detection and management of huge amounts of data, besides consideration of environmental effects on monitoring hazards and underground objects. Finally, this paper aims to help to solve the mentioned limitation through a comprehensive review of existing research.

## 1. Introduction

Deterioration of concrete structures and infrastructures, such as bridges and railways, is an ongoing challenge for the owners. Assessing the real condition of the deteriorated structures is important to identify types of defects on time, and make appropriate repair strategies [1,2,3]. Due to the large number of aging infrastructures, structural health monitoring (SHM) has achieved an efficient role in the operation and maintenance phase of structural life-cycle management. SHM facilitates controlling and assessing structural properties, therefore optimizing structural maintenance costs and increasing structural safety [4]. Structures have different responses under various loads during their life cycle which can be measured through SHM systems and sensors to obtain information of changed parameters and elements [5,6]. The main steps in health monitoring are structural observations and measurement, condition assessment, information management, planning and decision making, execution of repairs, assessment of repair and maintenance performance [7,8].

Speed and accuracy are two key factors in assessing the condition of constructions during health monitoring which will be heightened by using BIM programs. BIM has been shown to have enormous potential in benefitting the construction industry. BIM has an important role in making the SHM information accessible, practical and understandable. It could improve the quality of the assessment steps and facilities management or monitoring [9,10,11,12,13,14].

BIM includes tools, processes, and technologies for documenting and exchanging 3D digital models. BIM is a conventional basis for describing monitoring-related information because it prepares a methodology for interdisciplinary metamodeling to qualify various kinds of information from different fields [15]. Digital model-based BIM workflows facilitate the real-time evaluation of model changes. Effective data management is possible through linking elements of SHM systems such as sensors with external sources (e.g., sensor data stored in databases) for various aspects like monitoring states of building elements [15].

BIM processes are arranged depending on whether the processes are coordinated to the same software called “closed BIM” or to cross-software applications called “open BIM” [15]. The benefit of closed BIM processes is the harmony of models with the same file format, which is usually a dedicated file format of a particular software manufacturer. The closed BIM processes also have disadvantages such as some restrictions and the confined flexibility due to these restrictions. On the other hand, the Industry Foundation Classes (IFC) support platform-independent or open BIM processes. The IFC is standardized under ISO 16739-1:2018 in its current version, to describe and to exchange building information models. Through IFC, a standard for semantic models has been created describing building information in all stages of a life cycle of a structure [15]. The IFC follows an object-oriented approach, in which building information is considered as a set of objects and each object has attributes to describe it. Furthermore, the IFC provides a set of types, functions, and rules to obtain information properly to the area of interest for example structural engineering [15].

BIM can facilitate the control and monitoring process of the structures during the entire life cycle through the various stages of design, production of materials, selection of layout, and maintenance such as recycling and reuse of materials [16].

On the whole, the efficiencies of using BIM in SHM and maintenance are summarized as: management and control of SHM data, better interpretation by connecting real-time data in BIM models, and preparation of a confident database for various projects. While BIM programs and health monitoring methods are well known during the last decades, important questions remain considering usage and importance of building information models in health monitoring, including:

- What are the benefits of using high-quality visual programs in monitoring procedures?
- What are the new subject areas and innovative approaches related to monitoring with BIM?
- What are the limitations related to performance of BIM in monitoring process?
- What are the future agenda related to this field?

The current paper will answer to these important questions through a systematic review of BIM in monitoring and maintenance with the summarization of the relevant subject areas, presentation of the approaches of existing research, determination of the limitations of existing studies, and preparation of future research agenda. This study tries to prepare a suitable and efficient literature review about achievements of BIM in monitoring and maintenance to show improvements, limitations, research gaps, and future agenda and emerging technologies due to the rapid growth of this field.

## 2. Research Methodology

The current paper analyses all existing studies related to the performance of BIM in health monitoring with a focus on maintenance and monitoring processes in various fields from the beginning of the last decade until 2020 through both quantitative and qualitative approaches. In this study, the bibliometric analysis utilizes a quantitative method for checking the existing research and consists of five steps.

### 2.1. Keyword Search

A keyword search was conducted in the Scopus database with different keywords: (1) “Building Information Modelling”; (2) “Building Information Modeling”; (3) “BIM”; (4) “Building Information Model”; (5) “Health Assessment”; (6) “Health Monitoring”; (7) “SHM”; (8) “Structural Health Control”; (9) “Sensor”; (10) “Maintenance” and (11) “Structural monitoring”. This search resulted in 1700 articles.

### 2.2. Applying Filters

All 1700 selected articles were filtered according to the following limitations: (a) only journal articles, (b) published in English and (c) published from 2010 until 2020. This resulted in 601 articles.

### 2.3. Full-Text Analyses

A quick read of papers was then performed. This resulted in some papers being omitted for the following reasons: (a) they were related to other fields of study such as medicine and health (b) they were related to the design process whereas the subject under study was existing buildings and their monitoring during their life cycles. Note that although Scopus had subject area filtering, an individual investigation was conducted to identify all related articles. After this step, about 275 journal papers remained.

### 2.4. Web of Science (WoS) Verification Search

To overcome Scopus limitations and control the results, an individual search was done in Web of Science (WoS) with the same keywords, to check existing papers. The total number of journal papers increased to 280.

### 2.5. Categorizing Articles Based on Their Main Purposes

**Figure 1:**
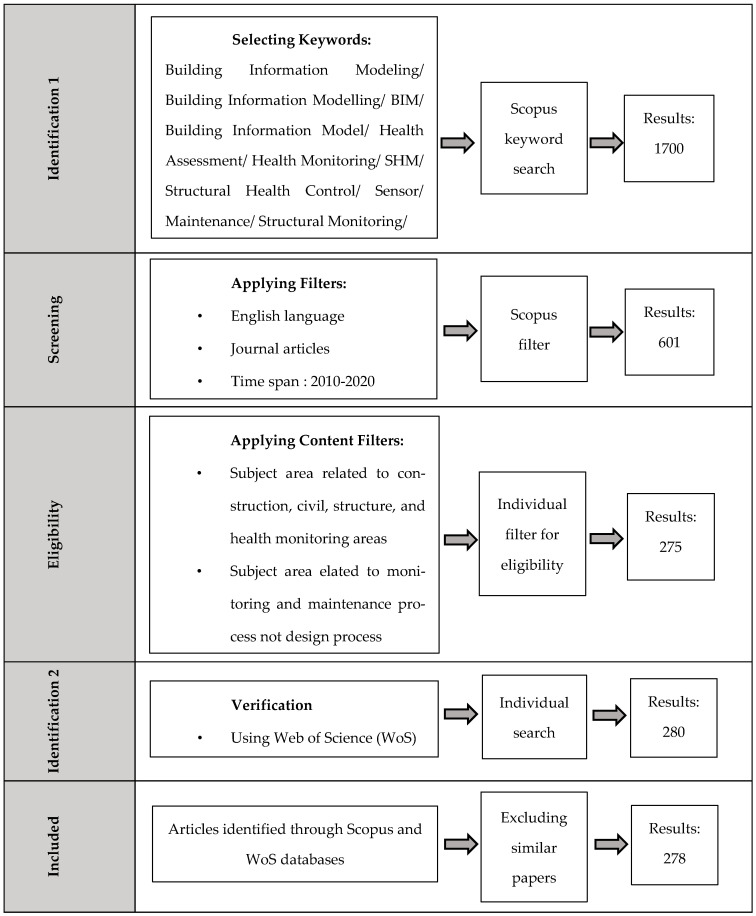
Methodology diagram of article selection for Building Information Modelling (BIM) in monitoring and maintenance.

The two search results were combined in one list by excluding literature reviews and duplicate articles, resulting in 278 articles that fitted our research subject. After reading the articles, they were categorized into various groups considering their subjects. As BIM usage in health monitoring is a recent topic for researchers, there is no comprehensive approach towards a specific subject. Therefore, relevant papers related to BIM in maintenance and monitoring steps were also reviewed. The PRISMA diagram of the systematic literature review (SLR) is shown in Figure 1.

## 3. Bibliometric Analysis

**Figure 2:**
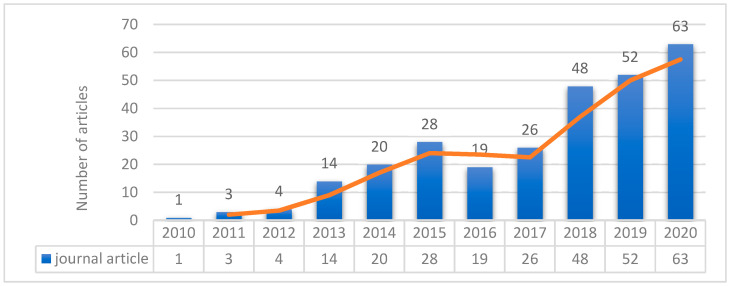
Published articles over the last decades related to BIM in monitoring and maintenance.

**Figure 3:**
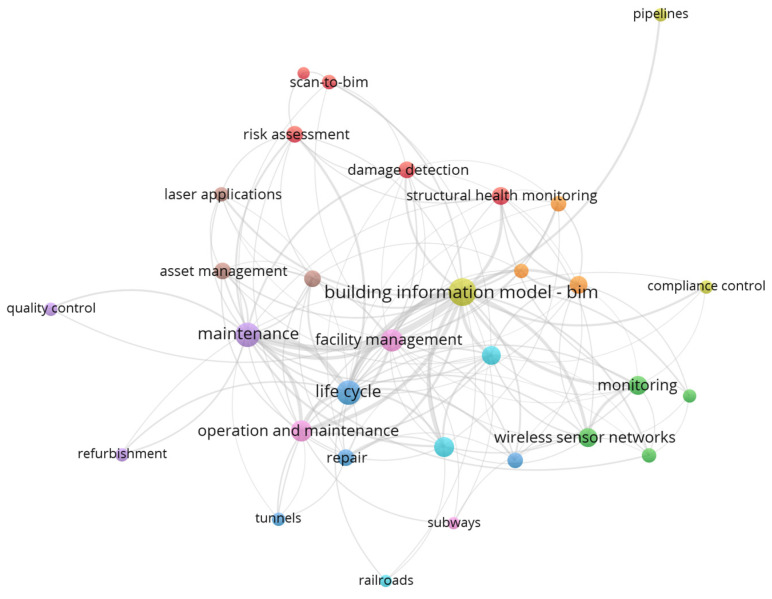
Frequency keywords clustering.

The bibliometric analysis showed continued growth in the rate of published articles about using BIM in the monitoring and maintenance process from 2010 to November of 2020, as shown in Figure 2. It shows that 92% of the papers were published in the last 7 years, indicating that much research has been devoted to this subject only recently. Based on our criteria, most articles in the field of our research, 48 in total, were published in the journal “Automation in Construction”. Other academic journals containing relevant papers are listed in Table 1. Building information modeling (BIM), maintenance, monitoring, and structural health monitoring (SHM) are the most frequently used keywords in the bibliometric analysis. This is followed by other keywords such as wireless sensor network, facility management and maintenance, repair, bridges, interoperability, and radio frequency identification, and risk assessment, internet of things, safety management, hazards, real-time, damage detection, compliance control, quality control, pipeline, laser applications and further keywords, as shown in Figure 3. This figure revealed that some keywords are main concepts such as monitoring or compliance control for structures and others are some case studies examined by researchers such as bridges, railways, pipelines, and others, therefore, the content analysis divided into two parts to consider all important concept related to our paper and all common case studies. These keywords’ repetition helped us in clustering the reviewed articles in some categories after combing the similar keywords [17]. The conceptual part includes (1) modeling and analysis which divided into two parts of modeling, standards and time-dependent analysis; (2) facility management (FM); (3) non-destructive testing with sensors; (4) diagnosing flaws and damages (also contains earthquake damages); and (5) dimensional compliance control, that mostly is for conventional buildings. The main case studies part includes (1) monitoring of offshore environment and pipeline (also contains mining and energy monitoring case studies); (2) monitoring of bridges and transportation facilities (also contains railway, tunnel, road, airports, highways, and utility tunnel case studies); (3) monitoring hazards (also contains fire, indoor safety, worker safety, environmental anomalies, and tower crane case studies); (4) various aspects of monitoring (contains case studies related to sustainability, safety, and refurbishment). Note that the clustering figure shows that many articles fall into the facility management and maintenance group. Overall, keyword analysis plays an important role in mapping articles but it is not sufficient for novel and total topics like BIM in monitoring and maintenance. Therefore, this content pattern was created based on clustering figures plus reviewing papers individually for preparing a clear and useful paper.

## 4. Content Analysis

**Figure 4:**
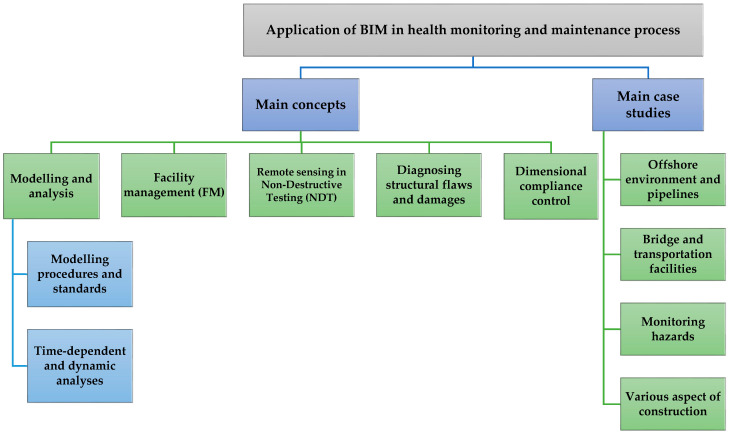
Results of content analyses.

By means of keyword clustering, the reviewed papers were categorized into groups based on their main purpose and modelling methodology. The dominant aims of each paper determined the category in which they were placed; however, some papers had the potential to be classified in alternate categories. At the end of each section, the forthcoming agenda related to the category was mentioned in order to facilitate future research. Figure 4 contains a summary of the content analyses and research paper subjects. Based on the research focus and contribution, the reviewed papers were categorized into some parts to cover our paper subject.

### 4.1. Main Concepts

This part aims to cover all concepts related to monitoring and maintenance with BIM. The goals of various studies and attitudes towards monitoring and maintenance are summarized in the following sections. At the end of each issue, the future works are mentioned for scientists to follow past studies.

#### 4.1.1. Modelling and Analysis Systems with BIM

##### Modelling Procedures and Standards

Based on explanation of introduction part, Industry Foundation Classes (IFC) is a standard for the interpretation of data of a building or facility during the construction or maintenance phase which is useful for categorizing data as a BIM model. IFC has been developed to enable various platforms integrate building information, i.e., interoperability enabling collaboration. The most common IFC format is known as a STEP file with the extension ‘.ifc’ [18]. All properties of buildings, components, and other parts of a structure are accessible through the BIM model. The BIM database is easily represented by the IFC standard for passing the exchange phase [18]. The IFC allows the construction process to be standardized since it is an open-exchange format and compatible with various applications [19]. Many researchers use BIM in SHM models to explain the benefits and limitations of current standards.

Rio et al. [19] tried to find a suitable way for saving SHM model data in digital databases through the BIM system since they believed that the extension of BIM standards was necessary for better performance in dynamic monitoring. A case study was considered to evaluate the applicability of the available IFC standard as a tool to have a 3D digital model of a real instrumented building. The interoperability of the model was also verified by using various modeling, viewing, and analysis software tools. Due to the different BIM requirements, software evaluation was designated an important aspect of all projects. Furthermore, researcher proposed kinematic sensors to fit the environmental sensors with their model; however, these sensors could not store the data without generic custom property sets.

Not all monitoring information for describing and exchanging building information models is supported by IFC; therefore, researchers have proposed an IFC schema extension to facilitate documenting SHM systems and managing the life-cycle changes [20,21].

Due to the limitations of previous studies, a formal basis has been prepared for monitoring information by using the IFC. The output information of this model proposes semantic combinations of health monitoring systems, topology conditions and relationships between elements of the health monitoring systems and structural systems in a well-defined format; however, this model does not have the potential of dynamic analyses [15]. Formal integration of health monitoring and control systems through the IFC schema into an IFC-compliant BIM model for cognitive buildings has also been considered [22]. An experimental and numerical examination was performed in creating a model for interpreting health monitoring and control systems and understanding relations of elements for better management and optimization of cognitive buildings. Some researchers have studied the maintenance approach of an IFC-based model and expanded it to time, cost, and performance through a step-by-step approach using BIM and radio frequency [23,24,25,26].

In the field of improving the visual properties of the monitoring process, researchers have tried different software and visual programming environment. Connecting environmental sensor data with BIM through Dynamo, Arduino, and Revit API, combining BIM with other tools like building maintenance management tools to gain comprehensive control over project monitoring and integrating BIM with geographic information system (GIS) for better management of building information have been done in this area [27,28,29]. Furthermore, some maintenance strategies have been done to manage massive data, visualization quality and data storage processes to serve as reference in other fields of study [30].

Researchers have studied various aspects of BIM approaches to amend the quality of designing and management of buildings by increasing speed and reliability in modelling big data with BIM, such as modelling of concrete structures and optimization of sensor deployment [31,32,33,34,35,36,37]. Using the 4D BIM approach for controlling concrete joints through Dynamo software reduced the inefficiencies of other approaches related to construction joints and planning pour despite some limitations such as problems related to circular elements, everyday fluctuating of concrete volume, and attendance of mixer trucks during the concreting process [38]. Of great interest is the plan for future studies to integrate 4D BIM models with on-site sensor monitoring to improve the health monitoring process visualizing and analyses, besides considering duration, productivity, and real-time hazards [39,40]. Since these upper dimension models increase the quality of constructions in all steps of costing design, maintenance, and monitoring, researchers are enthusiastic toward this field of study [41]. Other future agenda items in this category include: extending current IFC standards for better usage in health monitoring [20,21], interpretation of communication-related information through the IFC schema extension, expanding semantic models for covering more components of cognitive buildings [22], considering novel technologies such as closed-circuit television monitoring systems (CCTV), photovoltaic panels (PV), and VR in the IFC BIM- or RFID-based models [24], expanding interoperability solutions in the reinforcement supply chain [31], safety and quality management by 4D BIM model [33], application of BIM technology to task planning [34], dynamic modelling of pouring activities by collecting data from on-site sensors [38], and considering travel time on a road networks model for predicting concrete pouring duration [39].

##### Time-Dependent and Dynamic Analyses

Most of the studies that have been cited in various parts of this paper utilized real-time and dynamic monitoring; however, they have been categorized under those sections according to the main goal of each study [19,42]. After understanding the reliability of BIM for considering static loads, researchers through dynamic monitoring are able to diagnose the sensor data based on the last situation and changes in structural properties [43]. It helps researchers to better understand the structural performance, which results in confident decision making. Several papers exist in the field of structural monitoring systems in an interactive 3D environment [44]. Some researchers believe that using data-driven technologies involves problems such as lack of BIM approaches, the discrete AEC sector, and the lack of real-life practical examples; therefore, they have worked on parametric usage of BIM for structural monitoring with time-dependent sensor data and dynamic analysis in a high-quality 3D environment [23,45]. A bridge with an integrated fiber-optic sensor was considered as a case study to show that the model facilitates data interpretation through dynamic BIM environments. This was the result of research on health monitoring and solving its obstacles [45]. A knowledge gap related to time-dependent sensor data in building information models was addressed through studies utilizing such techniques as Domain-Specific Language (DSL) to reduce human effort in this process or coupling BIM model with a smart contract [46,47]. The combination of BIM and Internet of Things technology provided dynamic data transmission and appropriate data format to show the feasibility of this method in enhancing safety and quality in structures or equipment maintenance [48,49,50,51,52,53].

As stated before, traditional researchers utilized BIM mostly for design, monitoring, and analyses of the new structures during their lifetime; however, the perspective on BIM has changed so that it has recently been used to increase the quality of visualization for improving decision making [54,55,56]. Many kinds of research have had the problems of extracting data from sensors and the lack of interoperability, preventing the integration of BIM and SHM [54,57,58,59]. Previously, some researchers worked on using sensor output data in an IFC-based BIM model by using embedded sensors; however, Singh and Sadhu [56,60], conducted an online BIM model process to improve static BIM models from static to dynamic through real-time SHM data. Long-term monitoring of structures encountered real problems in the processing of large amounts of data that added to the BIM model; however, this issue was solved by using sensor data and handling applications to dynamic mode [61]. Analyzing a large volume of data was also facilitated through data compression techniques for solving data missing problems [62].

The future agenda in this field includes: New Spatial Design to Optimized Spatial Design [45], preparation of an interface for using ontology-based web services [58], mapping data maxima and minima, dashboard integration and various timeframes [63], considering prediction methods like a deep neural network (DNN) and convolutional neural network (CNN), and expanding the platform’s sensing for energy saving and lighting comfort.

#### 4.1.2. BIM and Facility Management (FM)

Managing data challenges in operation and maintenance (O&M) process of structures promote researchers to use BIM in facility management (FM) and combine it with other methods such as Data Mining (DM) to extract useful patterns and data, Performance Information Model (PIM) to obtain an integrated model, Thermal Infrared Sensing (TIS) to prepare a complete as-built BIM, a System Information Model (SIM) for asset management, IoT for helping decision making in facilities management, Quick Response (QR) codes or image algorithms for integration of facility information and repair process, and laser scanning or Radio Frequency Identification (RFID) for indoor localization of facilities [63,64,65,66,67,68,69,70,71,72,73]. For increasing the visualization quality in FM, the performance of a RESTful web application based on a BIM model was examined by researchers to show its benefits than traditional points of view [74]. Moreover, the efficiency of integration of the BIM technologies with real-time remote sensing tools on FM process was examined to show the progress of indoor maintenance monitoring and decision making during building life-cycle management [75].

Current research attitudes show increasing the continuous interest in Facility maintenance management (FMM) with BIM which covers over 65% of FM costs. Comprehensive studies were conducted in this area as using BIM with location-aware Augmented Reality (AR) to provide interaction among users and facilities, using Product-Service System (PSS) components in BIM models for managing equipment, combining BIM and IoT for lifecycle FMM, asset managements for improving the information processing, managing rapid-transit facilities such as subways and water treatment plants [69,76,77,78,79,80,81,82,83,84,85]. The remaining articles in this area will be considered in detail including BIM for monitoring subways, bridges, and other facilities. For controlling the maintenance work orders (MWOs) in FMM of indoor and outdoor components researchers also proposed cost-saving strategies to conduct this process automatically [86]. Moreover, considering risk actions in the FMM process is useful for maintenance policy which can be controlled through BIM and AR [87]. Many studies focused on healthcare facility management with some technologies such as AR or using 2D plans for creating BIM models and energy simulation which needs more examination related to modeling curved surfaces and envelope geometry [88,89,90,91,92,93]. Recently, the purpose of time-saving during the FM process for healthcare facilities, attracted scientists to utilize an algorithm through Natural Language Processing (NLP) to reduce human management problems [94].

Among all benefits, there are major limitations related to some projects which used BIM for FM, for example, some concentrated only on maintenance of certain systems or other has an unsuitable representation of maintenance problems in design review steps [95]. Considering IFC standard with specifications such as the Construction Operations Building information exchange (COBie) for reducing monitoring time, provided main problems in transferring and recording data or parameters in FM process and interoperability [96,97,98]. Also, some practical challenges restricted Post-Occupancy Evaluation (POE) data with BIM due to the problems related to changing FM models [99].

Previously, FM was not be considered in the design phase due to the lack of suitable modeling tools or access to facilities. These problems were solved by using a hybrid BIM-AR method for considering maintainability problems in design steps, integrating BIM with virtual reality (VR) and Microsoft Azure, using computer game software with BIM to help designers for saving time and cost and using BIM with digital programming to connect design and construction phase and energy analysis [95,100,101,102,103,104].

One of the purposes of using BIM in the maintenance and monitoring process is detecting faults which will be useful for the FM process through some Fault Detection and Diagnostics (FDD) algorithms to find damaged components such as heating or air conditioning equipment [105,106]. Many projects were performed to improve the FM-BIM methods for reduction of barriers in this field [107,108,109,110,111,112,113,114,115,116,117,118,119]. The remaining articles adopted different attitudes like conducting precise steps for integration of the required BIM-FM information for owners’ need, improving indoor navigation, refurbishment goals, using BIM for management of electrical, plumbing, and mechanical systems in a various way such as multi-scale BIM model, using BIM Perspective Definition (BPD) to increase reusability in systems, using mobile automated BIM-FM systems for solving problems related to transferring data and Improving task efficiency in FM process [120,121,122,123,124,125,126,127,128,129]. Moreover, interoperability challenges in the FM process solved by researchers by connecting BIM elements and FM information [130,131].

The future agenda in this category consists of improving the plug-in for using with different components models [129], reducing the impacts of environment on facility maintenance process [82], improve the PIM through using maintenance information of technical components [68], using various approaches to gather and share data for increasing automation among processes [83], considering the capacities of VR environment for reviewing designs [104], control energy conservation for mechanical, electrical, and plumbing systems [128], integrating BIM and VR technologies for controlling distance and time among sites [86], facility management with historical and current data of sensor technologies for controlling energy consumption [75], conducting various dataset to test NLP for FM [94].

#### 4.1.3. Sensors and Remote Sensing Technologies in Non-Destructive Testing (NDT)

Collecting real-time information of facilities, buildings and other construction-related fields is an effective way for managing construction operations which is accessible through sensing technologies. Wireless Sensor Network (WSN) is a practical technology for remote sensing purposes to control the inherent or environmental condition (temperature and humidity) during life-cycle management even in the non-structural field or hardly maintained cases than fixed monitoring systems [132]. Integrating WSN and BIM technologies increases the accuracy of monitoring hazards and energy-consuming that are main challenges during life-cycle management and human safety [133,134]. These tools mainly aim to reduce the maintenance cost of cognitive buildings by improving the accuracy of the decision–making based on collecting and processing data [22]. The performance of sensing technologies depends on the main purpose of monitoring and maintenance and is widespread among researchers for navigating equipment and construction safety by considering the advantages and disadvantages of each technology. Some important types are the global positioning system (GPS), encoder sensors, laser, Radio Frequency Identification Devices (RFID), Audio Technology, Radio Detection and Ranging (RADAR), magnetic sensors, Vision Cameras (VC) and also Ultra-Wide Band (UWB) [135,136]. Some sensing techniques that rely on radiation, sound, or electromagnetic signals are called Non-Destructive Testing techniques (NDT). They used for health monitoring such as acoustic emissions (AE) for detecting cracks of structures or laser scanning for damage detection in timber structures [137,138]. Integration of various sensors types is a useful approach for covering disadvantages of some sensors by other types for example the electromagnetic NDT sensor has geolocalisation problems which covers by using terrestrial laser scanning (TLS) and Structure-from-Motion (SfM) approaches or combining laser scanning and thermographic images technique is useful for vulnerability detection [139,140]. Developing an agnostic platform to link real-time data and static occupant data of sensors to BIM models, solving storage challenges due to WSN nodes movement, optimizing the sensor deployment for reducing dissipation of wireless energy, combining sensor data with BIM by Domain-Specific Language (DSL) technique showed suitable results in WSN activities [35,46,141,142]. Despite various efforts for atomization of remote monitoring with pressure sensors, recently researchers conducted a method for automatic generation of BIM models with economic sensors to increase accuracy, time-saving, and quality of modeling than TLS [135,143]. Moreover, the integration of multi-sensors performance and BIM laser lofting instrument for bridges, tall buildings, and tunnels is an innovative approach for preserving time and power in construction [144]. Many studies now exist in the field of remote sensing for monitoring which considers various types of sensors such as image recognition sensors, ultrasonic sensors network for crack detection, multimodal sensors, and others based on working conditions for different purposes such as vibration or emission monitoring [47,145,146,147,148,149,150,151,152,153,154].

The future work in this category involves testing multiple tower cranes navigation through sensors [136], increasing the intelligent functions of systems for gaining best algorithms based on the condition [132], increasing the Electromagnetic (EM) framework speed for sensing optimization [35], extraction of the information of sophisticated construction components with economic sensors [143], considering main effective factors on protective equipment misuse [135], examination various NDT sensing technologies such as spectroradiometer for fault detection [140].

#### 4.1.4. Using BIM for Diagnosing and Correcting Structural Flaws and Damages

Structural safety is the main goal of engineers all over the world; therefore, they are drawn to investigate novel ways of assessing structural defects, facilitated by BIM. Existing research in this area has focused on: goals such as conflict detection based on the life cycle versus as-built documents by considering all changes in the structure, fault detection of energy-consuming tools in buildings through BIM and simulation tools for energy monitoring, fault detection in buildings and non-structural components, damage detection in timber structures, tunnel flaws diagnosis to find maintenance strategies that are also used for utility tunnels, fault detection of hospitals for rehabilitation, automatic detection, and identification of lamps in a building [138,155,156,157,158,159,160,161,162,163,164,165]. Some papers used laser scanner data of existing structures along with BIM models [166]. Combination of BIM with radio frequency identification (RFID) facilitated fast identification of the surpassed strain spots as well as the controlling of structural elements and monitoring of structural performance for prefabrication (PC) projects in the BIM models [14,167].

BIM has a vast performance in modular construction as a suitable management tool and a powerful system, which can be improved by using real-time monitoring data for controlling structural damage [42,168,169,170]. It is effective for managing data and sensor-based elements of the SHM system in modular buildings and off-site construction [171]. Furthermore, visualization of damage in structural elements such as yielding or buckling through remote strain sensing and monitoring hidden structural elements are some benefits of BIM models.

Seismic monitoring is an important subject all over the world that is facilitated by using BIM modelling for controlling defects in structural and non-structural components [172,173,174]. BIM can improve the damage detection and rehabilitation process after an earthquake and improve cost estimation and time consideration in the upper dimension of post-earthquake BIM models [175,176].

Future research in this category would involve: large-scale sensor monitoring of modular buildings [171], fully mature and industry-grade fault detection for all devices of buildings [157], identifying component parameters and their interconnection structure in BIM models [158], a dynamic semantic extension mechanism and emergency response function for tunnels [165], statistical analysis of the differences in BIM tasks assigned to key activities [168], integrating Big Data technology to the Internet of Things-enabled BIM platform [170], upper dimension (5D) seismic repair analyses including structural and nonstructural components [176], laser scanning after damage and renovation of the defective BIM model based on damages [173], and integration different data sources for increased input accuracy [159].

#### 4.1.5. Dimensional Compliance Control and BIM

As dimensional incompliances prepared main problems for construction projects such as increasing cost and time, reducing safety and structural damages, accurate control is necessary during the construction and maintenance process. Many articles exist in this field that aims to promote the quality of control process through effective methods and BIM. Automated dimensional compliance control of in-place and precast components of concrete structure with terrestrial laser scanning, dimensional or geometric tolerances control of construction project, optimization of scan technologies for large-scale structures, managing LiDAR data by consideration of planar and linear properties, fabrication verification of pipes and flanges and compliance control of deep foundations, are main topics which utilized BIM as leverage for increasing the accuracy of their control phase [177,178,179,180,181,182,183,184,185]. The scan-to-BIM method is one of the popular subjects in this area that researchers considered a various aspect of it for dimensional control such as preparing main steps and criteria in the scan-to-BIM method, automatic deviation detection in the scan-vs-BIM process to reduce inspection’ time and cost, optimization of number and scan positions, facade damage detection with TLS, quality control with BIM for modular construction and controlling the surface flatness [186,187,188,189,190,191,192].

A future agenda for this category involves dimensional control of rebars in concrete structures with BIM [184], considering overlapping criteria with scan technologies and BIM [193], considering point cloud quality and BIM requirements in scan-to-BIM method [192], a new approach for identification of element boundaries [179], Sensitivity analysis of the effect of cylindrical isolator size in pipes and flanges [181], automatic identification of the key points for modeling [189], and conducting a suitable system for delivery and storage for cloud servers [180].

### 4.2. Main Case Studies

Besides using BIM in monitoring and maintenance of concrete and steel structures that extend to above categories, researchers have also used BIM for energy monitoring as well as monitoring other construction elements such as pipelines, offshore lighthouses, tunnels, airports, highways, subways, hazards, bridges, railways and other vital civil engineering elements, which are summarized in the following sub-chapters.

#### 4.2.1. Monitoring Offshore Environment and Pipelines

Recently, researchers have worked on using BIM models in novel ways such as for energy and asset monitoring; furthermore, most of the studies have been concentrated on land-based infrastructures such as buildings and bridges [133,194,195,196,197,198]. O’Shea and Murphy [18] explored health monitoring and asset management of offshore environments, concentrating on the design of an integrated structural health sensor network for an existing offshore structure. Output data and analysis results could be visualized coincidently by sending a static Revit model to a health monitoring tool for asset management. Their approach enabled parameters in the analysis of the structure to be controlled and visualized with BIM model and dynamic expanded SHM tool. Limitations of the existing models were related to the potential of interoperability with various BIM platforms. They also proposed two subjects for future research: (1) damage detection through the considering of sensor data; and (2) the visualization and assets management of a rich dataset BIM model.

Health monitoring through BIM is not restricted to structures and can be applied to practical fields such as underground pipelines [199]. Some researchers have focused on monitoring the corrosion of pipelines in the petroleum industry or the rehabilitation and maintenance process of pipelines [18,200]. Integrating a BIM system with passive sensors to collect site data by considering sensing technology has been a notably relevant approach [18]. Considering various types of pipeline material, improving the model by using novel sensing technology, and optimizing outputs by changing the location and angle of sensors are on the agenda within this category in the future [200].

#### 4.2.2. Monitoring Bridge and Transportation Facilities

After the collapse of the Tacoma Narrows Bridge in the US, bridge safety monitoring received widespread attention, and many bridges were equipped with monitoring sensors, such as Tsingma Bridge in Hong Kong, Sunshine Skyway Bridge in Florida, Akashi Kaikyo Bridge in Japan, and Hangzhou Bay Bridge in China. The efficiency of BIM systems encouraged researchers to use them for enhancing the quality of monitoring of bridges.

McGuire et al. [201] considered a bridge as a case study to reveal efficiency of using BIM in inspection and evaluation of bridges. Their method was successful in determining the location of deterioration and evaluating the load-rating factors or member capacities. Traditional approaches being insufficient in monitoring a huge number of health monitoring data, researchers utilized BIM for modeling of a bridge with long span to analyze a large amount of sensor data over a prolonged period. Their model was a simple, practical, and economic framework for verification and monitoring of long-term structure data [202]. Detecting and controlling the shape deformation of bridges prepares beneficial information for engineers during life cycle of bridges. Using BIM with light detection technology for monitoring bridges to reduce the risk of large deformation has been considered by researchers; however, it could not control large-scale scan data related to deflection and deformation maintenance and risk management [203].

There have been numerous studies related to the health monitoring and maintenance of bridges by using BIM models [204,205,206,207,208,209,210,211,212,213,214]. Combining BIM with other programs such as GIS improves the process of monitoring and risk or damage analysis [215,216]. Furthermore, using upper dimension BIM tools like 6D modelling through considering 3D model information along with cost, time and carbon footprint analysis improved maintenance decision making for traditional bridges [217]. In this field of study, researchers worked on expanding IFC-Railway to tunnel construction and sustainability evaluation of bridges [218,219]. Maintenance is an important aspect of transportation structures such as highways, roads, airports, and tunnels and can be expanded by applying different specific software packages with BIM [220,221,222,223,224,225]. Conducting maintenance strategies for highways or roads with BIM and sensor data provides optimization strategies during the life cycle of such infrastructures [226].

Like other fields of BIM in the monitoring process, managing a large amount of data or interchanging data in a model is very important and needs more studies especially in transportation infrastructures. In the field of bridge monitoring, BIM-based long-term monitoring, including monitoring of load, fatigue, steel corrosion, and concrete creep beside the study of optimization of sensor placement is important and needs further examination. Other items on the agenda in the future include sensor updating, data mining, longtime monitoring of bridges [210] and modelling structural and design complexity by considering variable parameters such as the length among expansion joints, properties of the superstructure, and the number of the expansion joints [206].

#### 4.2.3. Using BIM for Monitoring Hazards

Besides all the practical uses of BIM in health monitoring, integrating wireless sensor network data with BIM models creates a remote approach for controlling and monitoring hazards, eliminating and reducing the problems of locating people near hazardous environments during the inspection process by determining and controlling the exact location of each event in an emergency. There have been some limitations in these fields of study that need exact consideration: for example, increasing power supply capacity or introducing an additional power supply for gas detection components, and increasing the intelligent functions of the system for best performance in fire emergency scenes or low-dose hazardous gas exposures [227]. Various studies have been conducted related to using BIM to reduce risks in monitoring, such as integrating BIM for fire response operations, dynamic monitoring of fire after ignition, preserving the safety of equipment, fire prevention, BIM-framework for monitoring indoor environmental quality subway stations, combining BIM models and wireless sensor data for monitoring worker safety, building indoor safety management with Internet of Things (IoT) and BIM, monitoring thermal comfort with BIM, navigation systems of tower crane for blind lifts with BIM, supporting fire rescue methods, monitoring hazardous gas, controlling the hazards of working in confined space and real-time air dust monitoring with BIM for protection of workers [132,136,148,227,228,229,230,231,232,233,234,235,236,237,238,239,240,241].

The future agenda in this category includes estimating the effects of low-dose hazardous gas on human health [132], considering calibration function for each component of models [132], following the location of workers in construction environments through RFID & BIM [230], experimental testing of fire effects through various sensor prototypes [227], extension of frontline firefighters algorithm for use in entire complex buildings [148], experimental testing of using BIM in sensor-based tower crane systems [136], integrating BIM with artificial intelligence (AI) technologies for public environment management of smart cities [231], developing a procedure for modelling raw environmental and thermal data in BIM using Industry Foundation Classes (IFC) standards [235] and technical examination of dust monitoring in construction sites with BIM [238].

#### 4.2.4. Using BIM for Various Aspect of Construction Monitoring

Structural health monitoring has various fields that affect energy and cost-saving of buildings such as thermal transport, monitoring of thermal condition, air exchange, and moisture control [242,243]. The context of existing and future residential structures for hydro-thermal modelling and monitoring tools was another subject for health monitoring [244]. The advantages of utilization BIM for monitoring and optimization of complex refurbishment cases were the main subject of various studies that investigated the barriers and research directions of this approach [121,245,246,247,248,249]. Okakpu et al. [250] identified four dimensions that encompassed the impacts of BIM adoption specific to refurbishment projects and Becker et al. [248] conducted research for data collection and actual modelling. The concrete formwork process is the main part of construction management because any defects therein can lead to damage in structural elements; therefore, studies have considered BIM for concrete formwork design and monitoring by ignoring the maturity of concrete during the formwork time [251,252]. However, researchers have developed a method of BIM interoperability for controlling and monitoring concrete formwork problems [253]. This method reduced the required time before formworks could be removed by approximately 40%, by preserving concrete’s strength at a convenient level.

BIM technologies are used for various aspects of construction life-cycle management such as: assessing building performance for improvement requirements, monitoring all activities throughout the project, optimization of cost and time, using BIM for monitoring the sustainability of buildings with technologies such as IoT solution, developing a 5D BIM model for dynamic monitoring of deep foundation by considering time and cost, and real-time online monitoring of underground space to eliminate errors and difficulties related to this area [254,255,256,257,258,259,260,261].

Heritage building information modeling (HBIM) is another widespread research area that is practical for analyzing various case studies such as historical churches and masonry bridges by using active or passive sensors [262]. Researchers studied HBIM applied to masonry bridge through Revit commercial BIM software, asset management and maintenance of Cultural Heritage (CH), controlling sustainability and usability of CH through scanner laser and photogrammetry, conducting web application for historical building management, HBIM for storing life-cycle data through non-destructive testing and other aspects of these subjects for better analysis of heritage structures [263,264,265,266,267,268,269,270,271,272,273,274,275,276,277,278].

The future work in this category includes considering the inherent value of confined space monitoring system (CoSMoS), advanced methods for achieving patterns in real-time data for creating an intelligent digital built environment [243], BIM effects on application of life cycle assessment in buildings refurbishment [249] and development of intra-data collection to monitor local projects [251].

## 5. Conclusions

The current study prepared a systematic review related to BIM application in health monitoring and maintenance of structures. Generally, 278 papers published between 2010 and 2020 were selected. Limitations and improvements are presented, with a focus on maintenance and monitoring performance. Bibliometric and content examination of these articles revealed the main subject areas and resources wherein using BIM improved project results. Furthermore, this paper has tried to clarify future research attitudes for improving this field of study. However, further reviews or studies should consider the design process and its relationship between entire life-cycle model management through BIM. The bibliometric analysis revealed that most of the relevant articles were published in the Journal of Automation in Construction and 2020; these studies are associated with the benefits of using BIM in cost and time management. About 92% of the reviewed papers were published in the last 7 years, indicating that much research has been devoted to using BIM in monitoring and maintenance. The important findings of this beneficial review paper can be summarized as follows:

- In the content analyses based on keyword clustering and individual reviewing, reviewed articles can be divided into two groups. Some papers are highlighted based on their concepts and goals and others are based on their special case studies. Real-time monitoring and standards, diagnosing damages and dimensional compliance control, NDT with sensors and facility management were the main concepts. Main case studies related to bridges, pipelines, railways, tunnels, roads, airports, highways, and others considered controlling fire, indoor safety, worker safety, environmental anomalies, sustainability, safety, and refurbishment. Overall, total modeling problems such as extending IFC standards for exchanging data, managing the various type of sensing data and, interoperability among various BIM platforms were the main challenges among researchers in a wide range of studies that need more analytical and experimental work.
- Implementing some technologies such as laser scanning, radio frequency, real-time monitoring, closed-circuit television monitoring systems (CCTV), photovoltaic panels (PV), virtual reality, prediction methods such as a convolutional neural network (CNN) and deep neural network (DNN), enhanced the quality of monitoring and accuracy of decision making. However, increasing the intelligent functions of systems for gaining the best algorithms for all conditions, extraction of the information of sophisticated construction components with economic sensors, and examination of various NDT sensing technologies such as spectroradiometer for fault detection purposes remain challenges among researchers and should be followed in the future.
- The scan-to-BIM method is one of the popular subjects for dimensional compliance control to reduce inspection’ time and cost. Besides all improvement, considering point cloud quality and BIM requirements, element boundaries and overlapping criteria and delivery and storage for cloud servers are some gaps in this field.
- Combining the building information modelling process with other platforms such as GIS improves the process of monitoring and risk or damage analysis. Furthermore, using upper dimension BIM tools like 6D modelling through considering 3D model information along with time, cost, and carbon footprint analysis improves accuracy of this process. However, long-term monitoring, including monitoring of load, fatigue, steel corrosion, concrete creep and other environmental effects besides the study of mentioned programs is important and needs further consideration.
- The efficiency of integration of the BIM technologies with real-time remote sensing tools on FM process was examined to show the progress of facility maintenance monitoring and decision making during building life-cycle management. Considering risk actions in the FMM, time saving, and energy simulation in healthcare facility management, considering FM in the design phase, and using BPD to increase reusability in systems were some main improvements in this field. The limitation in using BIM for FM involves reducing the impacts of the environment on facility maintenance process, controlling energy conservation for mechanical, electrical, and plumbing systems, transferring and recording data or parameters in the FM process.
- Considering the novelty of this research field, there are many research gaps in all current studies; up to now, due to the lack of a standard for all modelling procedures, researchers have examined applications of BIM in monitoring through some assumptions. Extending the IFC schema, optimizing sensor data, and management of huge amounts of data are some major gaps, besides consideration of environmental effects on monitoring hazards and underground objects.

## Figures and Tables

**Table 1:** Journals with the most relevant article.

Journals	Total Article
*Automation in Construction*	48
*Sustainability*	11
*Sensors*	10
*Facilities*	9
*Buildings*	7
*Journal of Information Technology in Construction*	7
*Applied Sciences*	7
*Journal of Computing in Civil Engineering*	6
*Advanced Engineering Informatics*	6
*Journal of Performance of Constructed Facilities*	6
*Remote sensing*	5
*Advances in Civil Engineering*	3
*Smart Structures and Systems*	4
*Journal of Construction Engineering and Management*	3
*Structure and Infrastructure Engineering*	3
*Sustainable Cities and Society*	3
*KSCE journal of civil engineering*	3
*Journal of Engineering, Design and Technology*	3
*Visualization in Engineering*	3
*International Journal of Architectural Heritage*	3
*Engineering, Construction and Architectural Management*	3
*Journal of Architectural Engineering*	3

## Data Availability

No new data were created or analyzed in this study. Data sharing is not applicable to this article.
